# Tissue Tolerance Coupled With Ionic Discrimination Can Potentially Minimize the Energy Cost of Salinity Tolerance in Rice

**DOI:** 10.3389/fpls.2020.00265

**Published:** 2020-03-25

**Authors:** Koushik Chakraborty, Subhankar Mondal, Soham Ray, Pankajini Samal, Bhubaneswar Pradhan, Krishnendu Chattopadhyay, Meera Kumari Kar, Padmini Swain, Ramani K. Sarkar

**Affiliations:** ICAR-National Rice Research Institute, Cuttack, India

**Keywords:** chlorophyll fluorescence, leaf clip assay, salt stress, selective ion transport, sodium staining, transporters

## Abstract

Salinity is one of the major constraints in rice production. To date, development of salt-tolerant rice cultivar is primarily focused on salt-exclusion strategies, which incur greater energy cost. The present study aimed to evaluate a balancing strategy of ionic discrimination *vis-à-vis* tissue tolerance, which could potentially minimize the energy cost of salt tolerance in rice. Four rice genotypes, *viz*., FL478, IR29, Kamini, and AC847, were grown hydroponically and subjected to salt stress equivalent to 12 dS m^–1^ at early vegetative stage. Different physiological observations (leaf chlorophyll content, chlorophyll fluorescence traits, and tissue Na^+^ and K^+^ content) and visual scoring suggested a superior Na^+^-partitioning strategy operating in FL478. A very low tissue Na^+^/K^+^ ratio in the leaves of FL478 after 7 days of stress hinted the existence of selective ion transport mechanism in this genotype. On the contrary, Kamini, an equally salt-tolerant genotype, was found to possess a higher leaf Na^+^/K^+^ ratio than does FL478 under similar stress condition. Salt-induced expression of different Na^+^ and K^+^ transporters indicated significant upregulation of *SOS*, *HKT*, *NHX*, and *HAK* groups of transporters in both leaves and roots of FL478, followed by Kamini. The expression of plasma membrane and vacuolar H^+^ pumps (*OsAHA1*, *OsAHA7*, and *OsV-ATPase*) were also upregulated in these two genotypes. On the other hand, IR29 and AC847 showed greater salt susceptibility owing to excess upward transport of Na^+^ and eventually died within a few days of stress imposition. But in the “leaf clip” assay, it was found that both IR29 and Kamini had high tissue-tolerance and chlorophyll-retention abilities. On the contrary, FL478, although having higher ionic-discrimination ability, showed the least degree of tissue tolerance as evident from the LC_50_ score (amount of Na^+^ required to reduce the initial chlorophyll content to half) of 336 mmol g^–1^ as against 459 and 424 mmol g^–1^ for IR29 and Kamini, respectively. Overall, the present study indicated that two components (ionic selectivity and tissue tolerance) of salt tolerance mechanism are distinct in rice. Unique genotypes like Kamini could effectively balance both of these strategies to achieve considerable salt tolerance, perhaps with lesser energy cost.

## Introduction

Among all abiotic stresses, soil salinity is one of the major environmental constraints challenging crop production worldwide (Zhu, 2001). Gradual increase in the amount of sodium ion in the agricultural land is considered as an immense threat to global food security (Yamaguchi and Blumwald, 2005). Salinity affects nearly 0.8 billion hectares of land, which causes an annual loss of ∼27 billion US dollars worldwide (Qadir et al., 2014). Rice (*Oryza sativa* L.), a major cereal and staple food of two-thirds of the global population, is considered as a glycophyte. It shows significant growth retardation and yield loss beyond 3 dS m^–1^ of soil EC value (Munns and Tester, 2008). On an average, there is about 12% yield loss in rice with every dS m^–1^ rise in soil EC (Gao et al., 2007). However, the response is not universal, and some genotypes can withstand even 10–12 dS m^–1^ of salt stress, during early vegetative stage (Gregorio et al., 2002). Usually, rice plants show differential salt sensitivity at different stages of growth. It is particularly tolerant at germination stage and later parts of the vegetative stage but quite sensitive at early vegetative and beginning of the reproductive stages (Lutts et al., 1995; Zeng and Shannon, 2000).

Depending upon the duration of the stress, salinity can affect plants in two ways. Initially, it induces osmotic stress (independent of Na^+^ accumulation inside plants), which negatively affects water uptake by roots (Pardo, 2010; Roy et al., 2014). At later stages of stress, the ionic component, that is, Na^+^, becomes more dominant, causing necrosis and chlorophyll destruction (Roy et al., 2014). To cope with this, plants employ different strategies like exclusion of Na^+^ from cytosol to rhizosphere (ion exclusion) or compartmentalization of Na^+^ into vacuoles or other plant parts to avoid high Na^+^ accumulation in metabolically active tissues. This can also be achieved by maintaining the cellular ionic homeostasis by effective K^+^ retention (Munns and Tester, 2008). Previous studies reported genetic variations in rice for salt-tolerance strategies (Roy et al., 2014; Prusty et al., 2018; Chakraborty et al., 2019). The mechanism of ion exclusion is strongly associated with the expression of *SOS* pathway genes, where salt stress induces calcium signal by activating SOS2/SOS3 complex, which further activates SOS1, a plasma membrane Na^+^/H^+^ antiporter (Martinez-Atienza et al., 2007; Munns and Tester, 2008).

The influx of sodium strongly competes with activity of potassium channels/transporters present in the roots. These may be a salt-sensitive potassium transporter, *viz*., *AKT1* (Fuchs et al., 2005) or Na^+^-insensitive KUP-HAK group of plasma membrane K^+^/H^+^ transporter, *viz*., *HAK5* (Nieves-Cordones et al., 2010). On the other hand, the Class I type of HKT transporters, expressed in xylem parenchyma and phloem cells of both shoots and roots, help in xylem unloading of Na^+^ by selectively pumping out Na^+^ from xylem sap, thereby restricting its upward movement (Sunarpi et al., 2005; Møller et al., 2009). One of the important HKT family members, *SKC1* (often called *HKT1;5*/*HKT8*), is highly expressed in tolerant genotypes and helps to maintain a minimal Na^+^/K^+^ ratio in upper plant parts under salt stress (Ren et al., 2005). Such ion-exclusion strategies usually depend on active pumping out of Na^+^ against the concentration gradient; hence, more often than not, higher expressions of these ion pumps were reported to be associated with higher ATPases and pyrophosphatase activities (Mansour, 2014).

Apart from the phytotoxic effects of salt stress, accumulation of Na^+^ also induces osmotic stress. Exposure to salinity induces synthesis of organic osmolytes like proline, GB, and trehalose, which play a crucial role in counterbalancing Na^+^-induced reduction of water potential (Bohnert et al., 1995; Flowers and Colmer, 2008). Synthesis of such osmolytes via complex biosynthetic pathways not only utilizes huge C-skeleton but also incurs high energy cost. From ATP utilization’s point of view, biosynthesis of GB is the least-energy-intensive process, whereas biosynthesis of trehalose requires maximum energy (Dawood, 2016). Interestingly, under saline condition, elemental sodium itself can serve as an osmolyte and aid in maintaining the osmotic potential (Yeo and Flowers, 1986; Glenn et al., 1999; Chakraborty et al., 2016b). However, accumulations of high amount of sodium in metabolically active tissue are cytotoxic for glycophytes in general. Nevertheless, besides ion exclusion, tissue tolerance can also be considered as an effective strategy for salt tolerance (Niu et al., 2018b), which can be found in the plant naturally adapted to saline environment. These genotypes accumulate higher amount of sodium and preferentially compartmentalize them into vacuoles (Jiang et al., 2010; Fukuda et al., 2011). Compartmentalization of Na^+^ is also important to maintain structural integrity of chloroplast, mitochondria, and plasma membrane and to selectively distribute sodium in vacuole and cytosol for maintaining cellular homeostasis, water potential, and pH (Yamaguchi et al., 2001; Yoshida et al., 2009).

To date, most of the rice crop improvement program in the area of salinity tolerance focused on imparting ion exclusion or selective ion transport strategies to achieve a lower Na^+^/K^+^ ratio in physiologically active mesophyll tissues (Reddy et al., 2017). For this, tolerant donors like FL478, Nona Bokra, and Pokkali were used, all of which are known Na^+^ excluder and rely heavily on energy dependent active pumping out of Na^+^ (Reddy et al., 2017; Munns et al., 2019). But if some rice genotypes could be identified, which can effectively balance both ion-exclusion and tissue-tolerance strategies, then we may have dual advantages of (i) lesser energy cost for absolute Na^+^ exclusion and (ii) lesser cost of organic osmolyte production, as Na^+^ can also supplement as osmoticum. In reality, very few rice genotypes are known to possess traits like tissue tolerance and salt tolerance together. But our preliminary studies suggested that a genotype known as Kamini (originating from mangrove regions of Sunderbans, India) may possess both of these traits and effectively use them to achieve considerable salt tolerance, in spite of possessing higher Na^+^ concentration in aboveground parts than FL478. Therefore, the aim of our present study is to understand how these two mechanisms coexist in a single genotype and how these two strategies effectively balance each other, which might potentially minimize the energy cost of salt tolerance.

## Materials and Methods

### Plant Material and Growing Condition

For the present study, a hydroponic experiment was conducted in the net houses of ICAR-NRRI (National Rice Research Institute, Cuttack, India) with four rice *Oryza sativa indica* (FL478, Kamini, AC847, and IR29) genotypes having differential salt sensitivity. During the experiment period, the average temperature inside the net house ranged between 25.2 and 33.8°C, whereas the average relative humidity (RH) was moderate to highly humid, ranging between 65 and 88%. The light intensity inside the net house ranged between 720 and 1,050 μmol s^–1^ m^–2^. The genotype FL478 is a salt-tolerant line derived from Pokkali landraces, widely used as salt-tolerant check internationally, whereas IR29 is a released variety from IRRI (International Rice Research Institute, Manila, Philippines) used as susceptible check in salinity studies. Kamini, a local landrace collected from mangrove regions of Sundarbans, India, is known to possess an ability to thrive and give moderate yield under coastal saline areas. AC847 is another germplasm line selected for the present study.

For the hydroponic study, firstly, the seeds were preheated at48 ± 1°C for 5 days for breaking the dormancy and thensurface sterilized with 70% ethanol, followed by repeated wash with distilled water. The seeds were placed for germination in moistened paper in the Petri dishes and kept in the dark for 48 h to allow uniform germination. Two uniform pre-germinated seeds were placed onto each holes of floating Styrofoam panels having 10 × 10 holes, prepared as per the size of the tray used. The Styrofoam panels were placed in plastic trays (G.M. Polyplast, Pvt. Ltd., Mumbai, India) filled with Yoshida nutrient solution of pH 5.0 containing desired concentrations of macro-elements and micro-elements as described before (Gregorio et al., 1997). The plants were allowed to grow normally up to three- to four-leaf stage under constant monitoring of pH (5.25 ± 0.25) and EC of the nutrient solution every morning. Salt stress (in the form of NaCl solution) was imposed in one set of plants by transferring them onto a tray where required amount of NaCl solution (∼105 mM) was added to get an EC value of 12 dS m^–1^. The plants were kept at 6 dS m^–1^ of salt solution (∼53 mM of NaCl) for 2 days before imposing 12 dS m^–1^ of salt stress to avoid salt shock. Another set of plants was maintained as control and allowed to grow in normal Yoshida solution. The salinity treatment was continued until most of the plants of IR29 (susceptible check) reached a visual score of 9 (see Supplementary Figure S1 for visual manifestation of a score of 9).

### Visual Salt Injury and Standard Evaluation Score

Visual scoring of salinity stress symptoms was performed following SES in all studied genotype in a scale of 1 to 9 according to the standard protocol developed by IRRI (Gregorio et al., 1997). A score of 1 was given to plants that did not show any apparent sign of damage, whereas a score of 9 was given to plants that showed severe damage of shoot leading to complete bleach-out of greenness. The former was designated as highly tolerant, whereas the latter one designated as highly susceptible. Intermediate scoring was also given based on injury symptoms. The plant designated as tolerant, medium tolerant and susceptible based on a SES score of 3, 5, and 7, respectively (see Supplementary Figure S1 for visual manifestation of score). The SES scores were given to the plants, grown in five independent hydroponic trays maintained under control and stress conditions.

### Relative Water Content and Leaf Water Potential

Relative water content of the leaf was measured (Barrs and Weatherly, 1962) from five independent biological replicates of both control and stressed plants, where leaves were cut into pieces and weighed to collect the FW. Then it was placed on Petri dishes containing water for 24 h at room temperature and weighed to collect the TW. The same pieces of leaves were then oven-dried at 60°C for 72 h and weighed to measure the DW. Finally, RWC was calculated by the following formula:

RWC(%)=[(FW-DW)/(TW-DW)]×100.

Leaf water potential was measured from the leaf samples of five independent biological replicates from both control and stressed plants by placing small leaf disks onto the chambers of psychrometric water potential system (PSΨPRO water potential system, Wescor, United States) (Chen et al., 2012).

### Chlorophyll Content and Chlorophyll *a* Fluorescence Imaging

After imposition of stress, the total chlorophyll content was estimated daily from the second fully expanded leaf (from the top) of three independent biological replicates as per the method described by Arnon (1949). For this, 25 mg of chopped leaves was immersed in 10 ml of 80% acetone and kept at 4°C for 48 h. The chlorophyll *a*, chlorophyll *b*, and total chlorophyll content were measured by taking absorbance of the leaf extract at 645 and 663 nm in a double beam UV–Vis spectrophotometer (UV 2600, Shimadzu, Japan). The pigment contents were calculated as per the following formula:

Chlorophylla(μgml)-1=(12.7×ODat 663nm)-(2.69×OD⁢at⁢ 645⁢nm)

Chlorophyllb(μgml)-1=(22.9×ODat 645nm)-(4.08×OD⁢at⁢ 663⁢nm)

Totalchlorophyll(μgml)-1=(20.2×ODat 645nm)+(8.02×OD⁢at⁢ 663⁢nm)

Chlorophyll *a* fluorescence (ChlF) traits like maximum fluorescence (F_m_), efficiency of PS-II [Y(II)], and quantum yield of non-regulated energy dissipation [Y(NO)] were measured from the control and stressed leaves of hydroponically grown plants using an imaging fluorometer (IMAGING PAM-MAXI version, Heinz Walz GmbH, Germany) after 40 min of dark adaptation. The images for different ChlF parameters were captured and analyzed using Imaging Win v2.46i software supplied with the system. For each genotype × treatment combination, the leaf samples of three independent biological replicates were used for chlorophyll *a* fluorescence measurement. In each leaf, the measurement was done in three different positions by making uniform circular area [area of interest (AOI)] following the procedures described previously (Pradhan et al., 2018). Similarly, all the ChlF traits were also recorded from the leaf samples of tissue-tolerance assay following the same procedure.

### Estimation of Sodium (Na^+^) and Potassium (K^+^) Contents and Selective Transport of K^+^ Over Na^+^

Tissue sodium and potassium content was measured each day in roots and leaves samples of five independent biological replicates to study the accumulation pattern of different ions under salt stress. To measure the ion content, the fresh leaf samples were oven-dried at 60°C for a week, and 50 mg of dried tissue was crushed in a mortar and pestle with 25 ml of 1 N HCl. Tissue extraction was done with 1.0 N of HCl at 30°C for 48 h. Thus, obtained tissue extracts were then diluted and filtered using Whatman #40 filter paper. Finally, the amount of sodium and potassium was measured with the help of a flame photometer (PFP 7 flame photometer, JENWAY, United Kingdom). We also calculated the selective transport (ST) of K^+^ over Na^+^ (ST) by the following formula and the method described by Wang et al. (2005), where higher ST value indicates strong selectiveness and ionic discrimination present inside the plant during transport of ions to the upper parts of cells.

ST=(K/+Naratio+inshoot)/(K/+Naratio+inroot).

### Localization of Sodium (Na^+^) Ions Studied Through Fluorescence Sodium Indicator Dye

To visualize tissue-specific Na^+^ localization, we made thin sections of leaves and roots of both control and treated plants (subjected to 7 days of salt stress) from three independent biological replicates, which were previously fixed with 2.5% glutaraldehyde solution. Free-hand sections were stained with fluorescence sodium indicator dye (CoroNa Green AM, Invitrogen) and propidium iodide (Invitrogen) following the procedure as described by Marriboina et al. (2017). In brief, the sections were kept in 20 μM of CoroNa Green for 3 h, followed by staining with 2.5 μM of propidium iodide for 15 min before slide preparation and visualization under microscope. The cross sections of the leaves and roots were then visualized under laser scanning confocal microscope (Leica TCS SP5, Germany) and analyzed with Leica Advance Suite-AF software. The excitation and emission wavelengths were 492/516 nm for CoroNa Green and 493/636 nm for propidium iodide. To determine the level of Na^+^ fluorescence, we measured the intensity of the images for the area of about 7,973.3 μm^2^ and collect the reading in gray scale.

### Determination of Organic Osmolytes

The content of different core osmolytes, *viz*., proline, quaternary ammonium compound like GB, and trehalose, were measured from three independent biological and three technical replicates by different spectrophotometry-based methods. The proline content was estimated (Bates et al., 1973) from 500 mg of fresh tissues crushed in 10 ml of sulfosalicylic acid and filtered through Whatman #1 filter paper. Two milliliters of the filtrate was then mixed with 2 ml of acid ninhydrin and 2 ml of glacial acetic acid and boiled at 100°C for 60 min. The reaction was stopped by immediate cooling of the tubes in an ice bath. Finally, 4 ml of toluene was added to the reaction mixture and vortexed thoroughly. The absorbance of the upper toluene layer was measured with UV–Vis spectrophotometer (UV 2600, Shimadzu, Japan) at 520 nm, using toluene as blank.

The amount of GB was measured following the method described by Grieve and Grattan (1983), where 500 mg of dry samples was crushed in 10 ml of deionized water and kept at room temperature for 48 h before filtration with Whatman #1 filter paper. The filtrate was mixed with 1(N) H_2_SO_4_ in a 1:1 ratio and kept in an ice bath for 1 h. The 0.5 ml of thus obtained aliquot was mixed with 0.2 ml of cold potassium tri-iodide solution and stored in a freezer for 12 h. The samples were centrifuged at 10,000 rpm for 10 min at 4°C (Harmle Z32HK, Germany); and the collected pellet was dissolved with 9 ml of dichloroethane and stored at 4°C for 2.5 h. Finally, the absorbance was measured with a UV–Vis spectrophotometer (UV 2600, Shimadzu, Japan) at 365 nm. Trehalose content was estimated (Ferreira et al., 1997) from 10 mg of dried sample crushed with 2 ml of trichloroacetic acid (0.5M) and stored in a freezer for 20 min. The supernatant was collected after centrifuging it at 5,000 rpm for 10 min. The aliquot (0.3 ml) was mixed with 3 ml of anthrone reagent and heated at 100°C in a water bath. The samples were then immediately transferred to dark for 20 min. Finally, the absorbance was measured with UV–Vis spectrophotometer (UV 2600, Shimadzu, Japan) at 620 nm.

### Tissue-Tolerance Assay—Leaf Clip Method

*Ex situ* tissue-tolerance assay was performed with the same four (FL478, IR29, AC847, and Kamini) rice genotypes from three independent biological and three technical replicates to measure the pigment retention capacity of these genotypes under similar Na^+^ load in the mesophyll tissues. For this, leaves were collected from ∼25-day old normally grown plants, and the leaves were cut into pieces (∼10 cm) and placed in Petri dishes containing fresh and saline water (EC 12 dS m^–1^) for 7 days. The chlorophyll content, ChlF imaging, and tissue ion contents (Na^+^ and K^+^) were estimated as described above, on a daily basis, from both freshwater- and saline water-dipped sample. From the collected data, daily chlorophyll degradation rate and tissue tolerance (LC_50_ score represents a sodium concentration where half (50%) of the chlorophyll pigments were destroyed) was calculated for each genotype (Prusty et al., 2018).

### Gene Expression Analysis

Expressional analysis of key Na^+^ and K^+^ transporters and ion channels and H^+^ pumps in roots and leaves was performed by quantitative real-time PCR (qRT-PCR) analysis as per Vijayan et al. (2018). Briefly, total RNA was isolated from roots and leaves of control and stressed plants, 2 days after imposition of salt stress using RNeasy Plant Mini Kit (Qiagen). The extracted RNA was treated with DNase I to remove the genomic DNA contamination and checked in Nanodrop (ND 1000, Thermo, United States) and visualized in MOPS gel. About 1 μg of RNA was converted into cDNA using the Quantitech Reverse Transcription Kit (Qiagen) as per manufacturer’s protocol. Differential expressions of Na^+^/H^+^ transporters (*OsSOS1*, *OsSOS2*, *OsSOS3*, and *OsNHX1*), K^+^/Na^+^ transporters (*OsHAK5*, *OsHKT1;1*, *OsHKT1;5*, *OsHKT2;3*, and *OsAKT1*), K^+^ leakage channel (*OsROK*), and plasma membrane and vacuolar H^+^ pumps (*OsAHA1*, *OsAHA7*, *OsV-ATPase*, and *OsV-PPase*) were studied (see Supplementary Table S1 for primer details). Gene-specific primers were designed from CDS (Coding Sequence) by using the QuantPrime software^1^ (Arvidsson et al., 2008). For quantitative real-time PCR, we used QuantiFast SYBR Green PCR reaction kit (Qiagen) and amplified it using QuantStudio 5 Real time PCR (Applied Biosystems, Thermo). Three biological and two technical replications were used in qRT-PCR to amplify the genes in control and stressed conditions, where relative expression level was checked by comparing the level of expression of gene with control using 2^–Δ^ ^Δ^ ^CT^ method (Livak and Schmittgen, 2001). In every reaction, we used *Os18S_rRNA* gene as an internal control to normalize the PCR.

### Statistical Analysis

All the data recorded were the mean values ± standard error (mean) of at least three independent replications. The experiment was conducted in two-factor completely randomized design, and the data were subjected to two-way ANOVA as per the experimental design using SPSS (version 16.0) software. The ANOVA found significance for treatment × genotype interaction at 5% level of significance.

## Results

### Effect of Salt Stress on Plant Survival, Chlorophyll Pigment System, and Biomass

**FIGURE 1:**
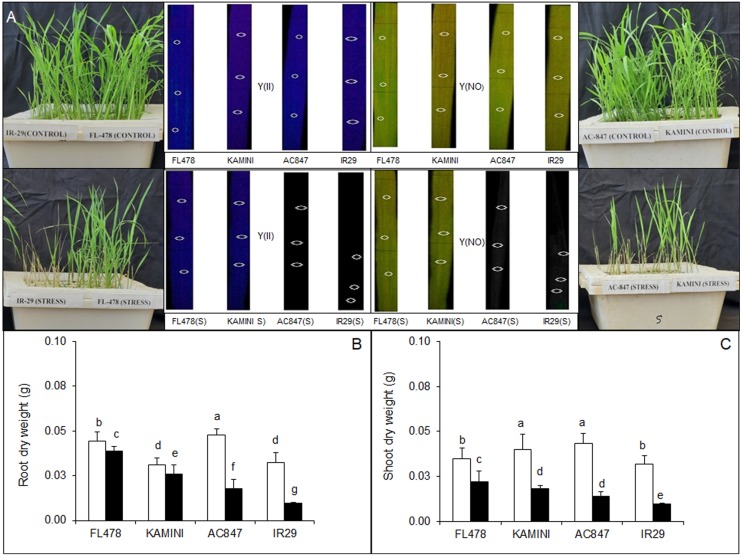
Phenotypic variations **(A)** and effect of salt (12 dS m^–1^) stress on DW of root and shoot **(B,C)** in four rice genotypes subjected to 7 days of stress in hydroponic assay. The values presented are the mean ± SE of five independent biological replications and values sharing the same letter for each treatment × genotype combination, are not significantly different (*P* ≤ 0.05) according to Tuckey’s test.

In the present study, four rice genotypes (FL478, Kamini, AC847, and IR29) showed differential salt sensitivity when subjected to 12 dS m^–1^ of salt stress for 7 days (Figure 1A). In fact, the genotypes started to show phenotypic variations just after 3 days of stress imposition. Based on the visible salt induced injury, relative tolerance of the genotypes was worked out by assigning SES to each genotype 7 days after imposition of stress. Both FL478 and Kamini got a score of 3, suggesting a high degree of tolerance in these genotypes, whereas IR29 was the most susceptible genotype with a score of 9 and AC847 had a score of 7. The total chlorophyll content (recorded daily since imposition of salt stress) showed faster chlorophyll degradation in susceptible genotypes like IR29 and AC847. The total chlorophyll content reduced from 2.23 to 0.89 and from 2.24 to 0.91 mg g^–1^ in IR29 and AC847, respectively. Interestingly, there were no significant reductions in total chlorophyll content in either FL478 or Kamini during the stress period (Supplementary Figure S2). The loss of integrity of the photosystem II was lesser in FL478 and Kamini as compared with AC847 and IR29. The values of Y(II) were 0.78 for FL478 and 0.59 for Kamini, whereas Y(NO) values were 0.21 for FL478 and 0.40 for Kamini under stress (Figure 1A).

Significant reduction in both root and shoot biomass was observed in susceptible genotypes like IR29 and AC847 (Supplementary Figure S2). Maximum reduction in root FW was observed in IR29 (46%), followed by AC847 (35%), whereas it was much less in Kamini (22%) and almost no reduction in FL478 (Supplementary Figure S2). Salinity-induced reduction in shoot FW was the highest in AC847 (83%), followed by IR29 (80%), Kamini (50%), and FL478 (42%). Based on DW data, it was observed that maximum biomass reductions were in the order of IR29 (69%, shoot; 70%, root) > AC847 (67%, shoot; 62%, root) > Kamini (55%, shoot; 17%, root) > FL478 (37%, shoot; 12%, root), which correlated with our visual scoring data as well (Figure 1). The results clearly showed that the effect of salt stress was much pronounced in shoot than in root. The root and shoot lengths also showed significantly higher reduction in IR29 and AC847 as compared with FL478 and Kamini (Supplementary Figure S2).

### Effect on Ion Homeostasis (Na^+^ and K^+^ Contents; Na^+^/K^+^ Ratio)

Salinity stress altered the ionic composition in both roots and shoots. Day-wise increment in total root Na^+^ content was almost similar in all the four genotypes. At the end of 7 days of stress, the highest Na^+^ accumulation was observed in roots of FL478 (674 mmol g^–1^ DW), followed by 642, 628, and 574 mmol g^–1^ DW for AC847, Kamini, and IR29, respectively. Unlike root, a clear-cut difference in Na^+^ accumulation was observed in leaves (Supplementary Figure S3). Here, the accumulation of Na^+^ was the least in FL478 (558 mmol g^–1^ DW), followed by Kamini (798 mmol g^–1^ DW), whereas it was ∼1,100 mmol g^–1^ DW in both IR29 and AC847.

**FIGURE 2:**
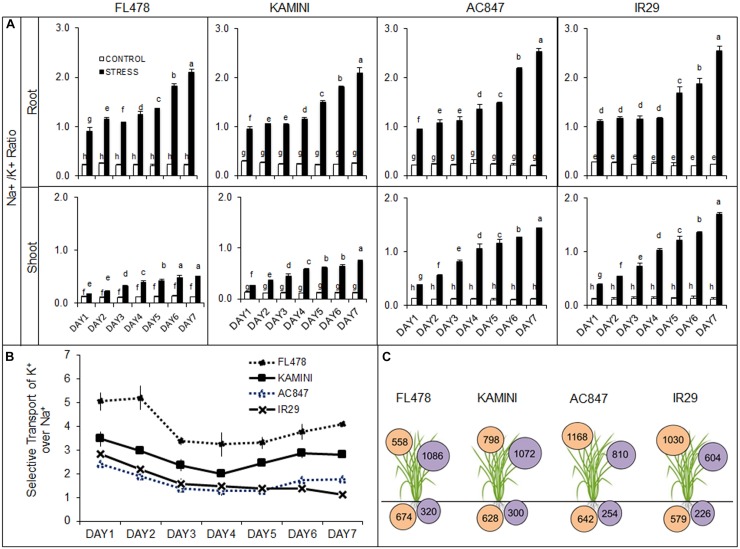
Effect of salt (12 dS m^–1^) stress on day-wise changes in root and shoot Na^+^/K^+^ ratio **(A)**, selective transport of K^+^ over Na^+^ from root to leaves **(B)**, and final Na^+^ (in mmol g^–1^ DW inside orange circle) and K^+^ (in mmol g^–1^ DW inside purple circle) accumulation in root and leaves at the end of 7 days of stress **(C)** in four rice genotypes subjected to 7 days of stress in hydroponic assay. The values presented are the mean ± SE of five independent biological replications and values sharing the same letter for each treatment × genotype combination, are not significantly different (*P* ≤ 0.05) according to Tuckey’s test.

Unlike root Na^+^ content, we found significant differences in root K^+^ content in studied genotypes under salt stress (Supplementary Figure S3). After third day of stress imposition, rapid decline in root K^+^ content was observed in IR29 and AC847. Finally, after 7 days of NaCl stress, the highest root K^+^ content was observed in FL478 (320 mmol g^–1^ DW), followed by Kamini (300 mmol g^–1^ DW), AC847 (254 mmol g^–1^ DW), and IR29 (226 mmol g^–1^ DW). The differences were more prominent in leaf K^+^ content (Figure 2 and Supplementary Figure S3). Both FL478 and Kamini did not show any significant change in leaf K^+^ content, but it showed considerable drop in IR29 and AC847. More than 40 and 30% reduction in leaf K^+^ content was observed in IR29 and AC847, respectively, after 7 days of salt stress. Significant changes in Na^+^/K^+^ ratios were observed in both leaves and roots. Owing to better K^+^ retention in root, a Na^+^/K^+^ ratio remained ∼2.0 in both FL478 and Kamini, but it was more than 2.5 for IR29 and AC847 at the end of 7 days of stress period (Figure 2A). The least Na^+^/K^+^ ratio was observed in the leaves of FL478 (0.51), followed by Kamini (0.75), whereas it was 1.70 and 1.45 in IR29 and AC847, respectively.

### Selective Transport of K^+^ Over Na^+^ From Root to Shoot in Response to Salt Stress

The ionic imbalance that occurred in roots and leaves in response to salt stress had given us an estimate of differential ST ability of K^+^ over Na^+^ (ST) from root to shoot in these genotypes. The genotype FL478 showed a strong ionic discrimination for upward transport of Na^+^ from root to shoot throughout the stress period (Figure 2B). An ST value of 4.1 after 7 days of stress indicated that FL478 preferentially transported K^+^ over Na^+^ even under higher external Na^+^ load. Susceptible genotypes like IR29 and AC847 recorded an ST value of 1.1 and 1.8, respectively, suggesting their inability of ionic discrimination. Kamini having an ST value of 2.8 appeared to be moderately selective in upward transport of ions under salinity stress.

### Tissue Localization of Na^+^ Visualized Through Confocal Microscopy

**FIGURE 3:**
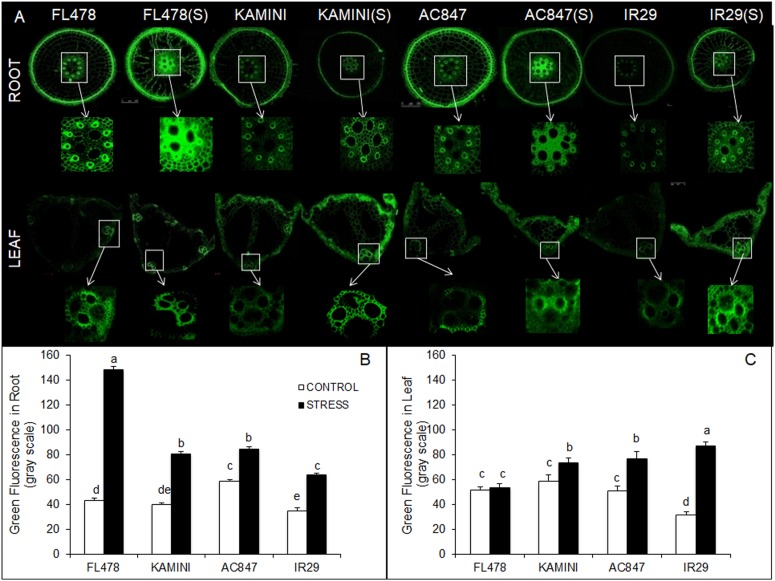
Cross section of root and leaf tissues stained with CoroNa Green fluorescent dye of the four rice genotypes subjected to 7 days of salt stress (12 dS m^–1^) in hydroponic assay **(A)**. Green fluorescence intensity of root **(B)** and leaf **(C)** sections from control and treated samples measured in gray scale. The values presented are the mean ± SE of at least three independent biological replications and values sharing the same letter for each treatment × genotype combination, are not significantly different (*P* ≤ 0.05) according to Tuckey’s test.

Further, to study the tissue-specific localization of Na^+^, we performed Na^+^-specific fluorescence staining of root and leaf tissues to visualize under confocal microscope. The cross sections of root and mesophyll tissues stained in CoroNa Green and propidium iodide dye revealed differential Na^+^ accumulation near vascular bundles of roots and leaves (Figure 3A and Supplementary Figures S4, S5). Here, the intensity of green fluorescence was directly proportional to the sodium content present on a particular plant part. The data obtained from the images clearly showed that FL478 maintained high amount of sodium, near the vascular bundles of root tissue showing a relative green channel fluorescence intensity of 148.72 units. The fluorescence intensities near the vascular bundles of roots were quite less in other genotypes (84.36, 81, and 64 units in AC847, Kamini, and IR29, respectively) (Figure 3B). But unlike the root tissue, in the mesophyll tissue, the scenario was almost opposite, where the highest green fluorescence intensity was observed in IR29 (87.07), followed by AC847 (76.95) and Kamini (73.47). FL478 did not show any increase in green fluorescence in the mesophyll tissue, which reaffirmed the least Na^+^ accumulation potential of this genotype in upper plant parts (Figure 3C).

### Plant Water Status and Production of Organic Osmolytes in Response to Salt Stress

**FIGURE 4:**
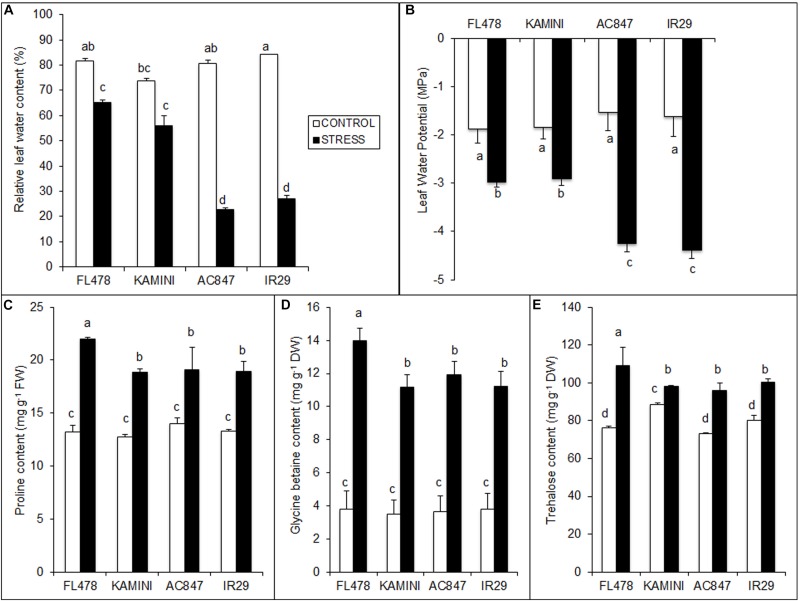
Effect of salt (12 dS m^–1^) on RWC **(A)**, LWP **(B)**, organic osmolyte contents, *viz*., proline **(C)**, GB **(D)**, and trehalose **(E)** in four rice genotypes subjected to 7 days of stress in hydroponic assay. The values presented are the mean ± SE of at least three independent biological and three technical replications and values sharing the same letter for each treatment × genotype combination, are not significantly different (*P* ≤ 0.05) according to Tuckey’s test.

Plant water status measured through RWC and LWP showed significant differences between the studied genotypes at the end of 7 days of stress period (Figures 4A,B). Drastic reduction in RWC in IR29 (from 84 to 27%) and AC847 (from 81 to 23%) was observed, whereas the decrease in FL478 and Kamini was less (from 82 to 65% in FL478 and from 74 to 56% in Kamini) (Figure 4A). Similarly, the LWP showed the highest drop in IR29 and AC847, where it reduced to −4.39 and −4.25 MPa, respectively, whereas it reduced to only −2.98 and −2.95 MPa in FL478 and Kamini, respectively (Figure 4B).

Significant variation in salinity-induced organic osmolyte production in the studied genotypes was observed in the present study (Figures 4C–E). The content of proline, GB, and trehalose was particularly higher in FL478 as compared with that of other three genotypes after 7 days of stress imposition. The highest free proline content was observed in FL478 (22 mg g^–1^ FW), which was significantly higher than that in IR29, Kamini, and AC847. Similarly, the contents of GB and trehalose were higher in FL478 (>3.7-fold increase for GB and ∼1.5-fold for trehalose) than the rest of the genotypes. On the contrary, the contents of these metabolites were at par in Kamini and susceptible genotypes such as IR29 and AC847.

### Effect of Salt Stress on Tissue-Tolerance Ability (Tested Through Leaf Clip Assay)

**FIGURE 5:**
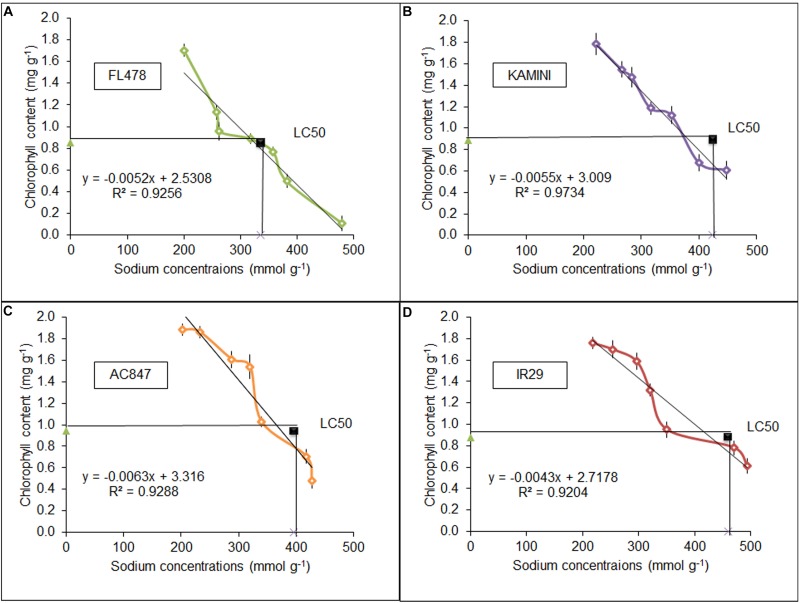
Tissue-tolerance score of FL478 **(A)**, Kamini **(B)**, AC847 **(C)**, and IR29 **(D)**, measured in LC_50_ value calculated from the leaf clip assay in four different rice genotypes under 12 dS m^–1^ of salt stress. The values presented are the mean ± SE of at least three independent biological and three technical replications.

Tissue tolerance of the individual genotypes was estimated *ex situ* by leaf clip assay. Day-wise chlorophyll degradation of these leaf clips was estimated along with daily uptake of Na^+^ in four studied genotypes for 7 days of stress period. Surprisingly, the highest rate of chlorophyll degradation was in FL478, where the total amount of chlorophyll reduced from 1.705 to 0.11 mg g^–1^ after 7 days (Figure 5). On the contrary, both IR29 (1.76–0.61 mg g^–1^) and Kamini (1.78–0.608 mg g^–1^) showed the least chlorophyll degradation over the stress period. This result was completely opposite to our hydroponic assay, where we found almost no change in chlorophyll content in FL478 but the highest reduction in IR29. This suggests that in order to understand the actual tissue-tolerance potential of the genotypes, it is necessary to nullify the ionic-discrimination barrier so that equal amount of Na^+^ is allowed to enter the mesophyll tissues. Further, we calculated the tissue-tolerance value on the basis of LC_50_ score, where this score represents the amount of Na^+^ required to reduce the initial chlorophyll content to half. Salt-tolerant genotype FL478 had the least tissue-tolerance score (LC_50_) of 336 mmol g^–1^, whereas genotypes like IR29 and Kamini had better tissue-tolerance score (LC_50_ = 459 and 424 mmol g^–1^, respectively) (Figures 5B,D).

### Changes in Chlorophyll *a* Fluorescence: Hydroponic *Vis-à-Vis* Leaf Clip Assay

**FIGURE 6:**
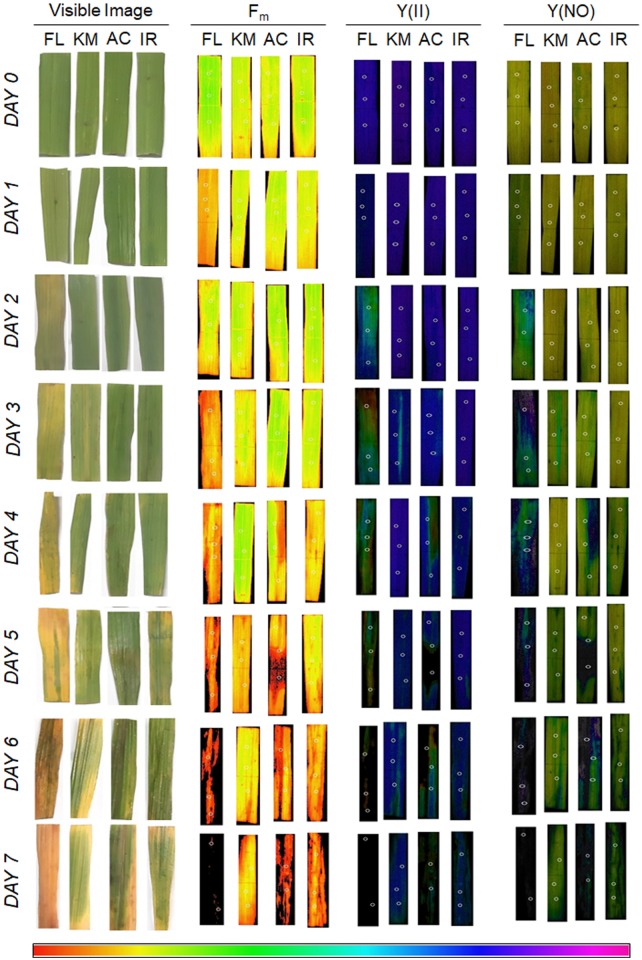
Day-wise visible and fluorescence images [F_m_, Y(II), and Y(NO)] of leaves from leaf clip assay of four rice genotypes subjected to 12 dS m^–1^ of salt stress, where FL:FL478; KM:Kamini; AC:AC847; and IR:IR29. The images shown here are a typical representation of at least three biological replications.

**FIGURE 7:**
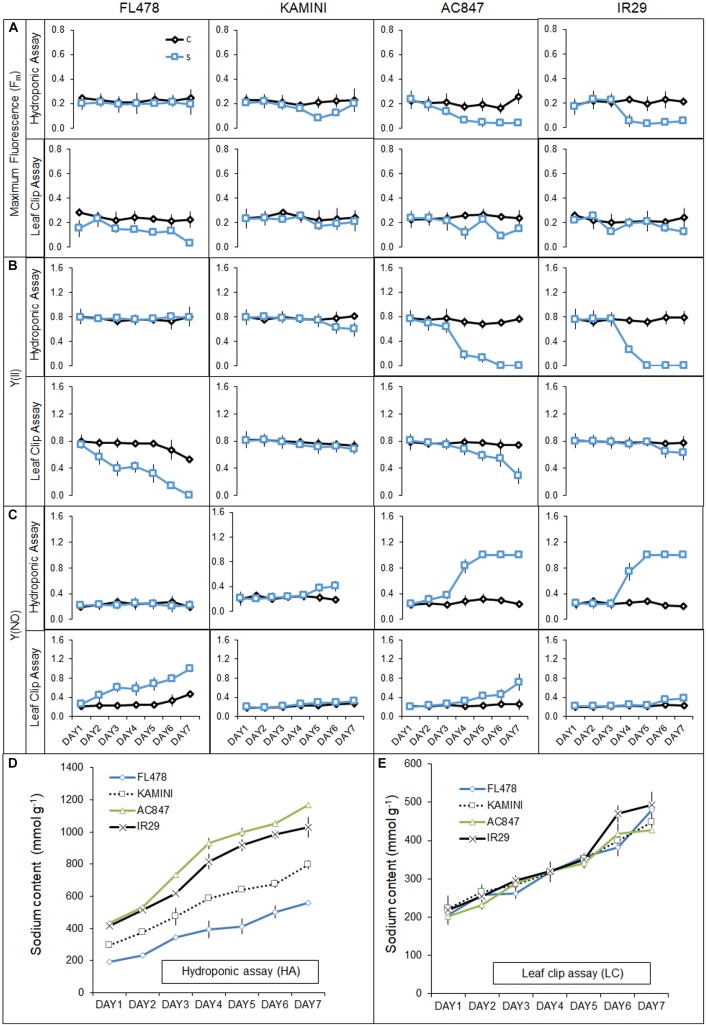
Effect of salt stress (12 dS m^–1^) on different chlorophyll fluorescence traits, *viz*., F_m_ **(A)** and Y(II) **(B)** and Y(NO) **(C)** of four rice genotypes subjected to stress in both hydroponic (HA) and leaf clip (LC) assays. Day-wise uptake of leaf Na^+^ content of four rice genotypes in hydroponic (HA) assay **(D)** and leaf clip (LC) assay **(E)**. The values presented are the mean ± SD for **(A–C)** and mean ± SE for **(D,E)** of at least three independent biological and three technical replications.

In the present study, our hydroponic and leaf clip assay reflected contrasting behavior of tissue-tolerance and overall salt-tolerance abilities of the studied genotypes (Figure 6). FL478, the most salt-tolerant genotype from hydroponic assay, was proved to have the least tissue-tolerance ability when exposed to leaf clip assay. Genotypes like IR29 and AC847 showed a sharp decline in different chlorophyll fluorescence parameters, *viz*., maximum fluorescence (F_m_), efficiency of PS-II [Y(II)], and quantum yield of non-regulated energy dissipation [Y(NO)] from third day onwards in hydroponic assay. In IR29 and AC847, F_m_ reduces from 0.215 (control) to 0.05 (stress) for IR29 and 0.257 (control) to 0.04 (stress), respectively, whereas Y(II) dropped from 0.791 (control) to 0.00 (stress) for IR29 and from 0.766 (control) to 0.00 (stress) for AC847. On the contrary, genotypes like FL478 and Kamini showed no significant drop in any of these parameters until 5 days of stress in hydroponic assay, which indicated relative superiority of these genotypes for their chlorophyll pigment integrity and photosynthetic rate retention ability under saline condition. Interestingly, when leaves (from control treatment) of these four genotypes were subjected to 12 dS m^–1^ of NaCl stress *ex situ* in leaf clip assay, almost an opposite trend in their ability to tolerate salt stress was noticed. Here, no significant change in F_m_, Y(II), and Y(NO) were observed in IR29 even after 5 days of salt stress (Figure 7). The reduction in maximum fluorescence (F_m_) was the highest in FL478 (from 0.223 to 0.031) in leaf clip assay, whereas it did not change at all in Kamini and dropped slightly in IR29 after 7 days of stress imposition. To understand such contrasting responses of the studied genotypes in hydroponic and leaf clip assay, we estimated the day-wise net Na^+^ uptake of the leaf clips and compared it with the day-wise Na^+^ accumulation of the leaves in hydroponic assay (Figures 7D,E). As mentioned earlier, owing to high ionic-discrimination ability present in FL478, lesser Na^+^ could reach the mesophyll tissue in this genotype during *in situ* uptake of Na^+^ during hydroponic assay. But to nullify this effect when leaf clips of individual genotypes were directly exposed to 12 dS m^–1^ of NaCl stress, we found almost similar mesophyll tissue Na^+^ content in every genotype. Our results suggested that a genotype like Kamini performed well in both hydroponic and tissue-tolerance assays, which is very unique among the studied genotypes.

### Changes in the Transcript Abundance of Different Na^+^- and K^+^-Specific Transporters/Ion Channels/Pumps

**FIGURE 8:**
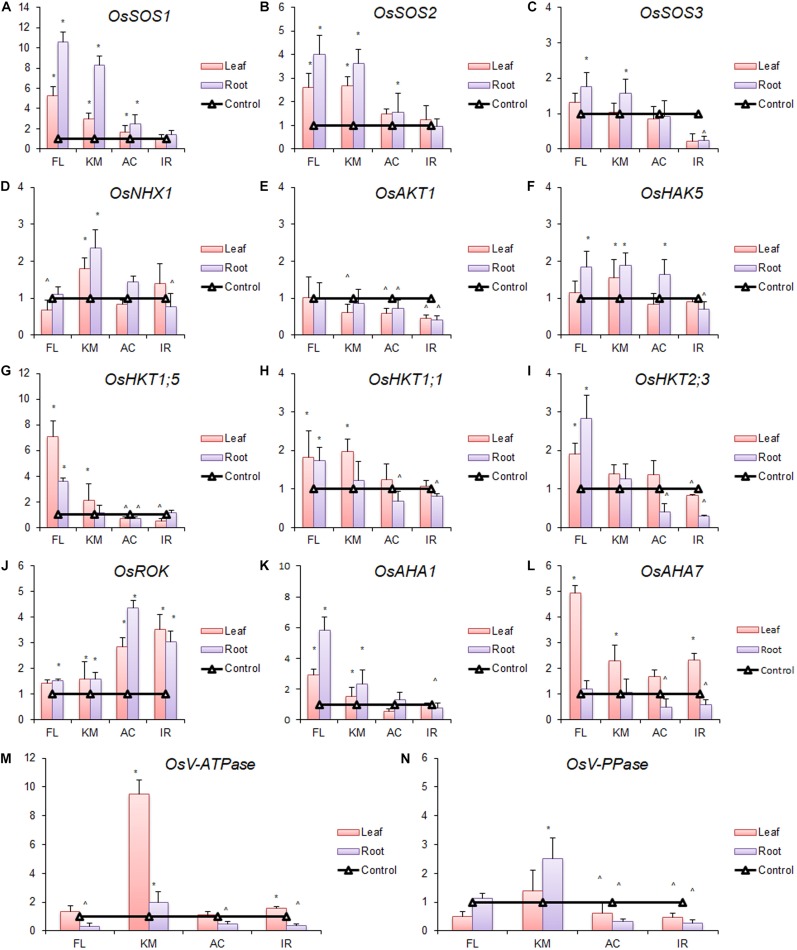
Changes in expression profile (relative expression in terms of 2^–Δ^ ^Δ^ ^CT^ fold change) of different Na^+^/H^+^ transporters, *viz*., *OsSOS1* **(A)**, *OsSOS2* **(B)**, *OsSOS3* **(C)**, and *OsNHX1* **(D)**; different K^+^/Na^+^ transporters, *viz*., *OsAKT1* **(E)**, *OsHAK5* **(F)**, *OsHKT1;5* **(G)**, *OsHKT1;1* **(H)**, and *OsHKT2;3* **(I)**; *OsROK* **(J)**; plasma membrane H^+^ pumps, *viz*., *OsAHA1* **(K)** and *OsAHA7* **(L)**; and vacuolar H^+^ pumps, *viz*., *OsV-ATPase* **(M)** and *OsV-PPase* **(N)** in roots and leaves of four rice genotypes subjected to 2 days of salt stress (12 dS m^–1)^, where FL:FL478; KM:Kamini; AC:AC847; and IR:IR29. * and ^ denote significant upregulation and downregulation, respectively, in comparison with control. The values presented are the mean ± SE of three independent biological and two technical replications.

In the present study, metabolic alterations in terms of transcript abundance of different Na^+^- and K^+^-specific transporters/ion channels/pumps were noticed in studied genotypes in response to salt stress (Figure 8). Significant upregulation of expression of key transporters was observed in FL478 and Kamini in response to salt stress. The expression of *OsSOS1* gene was upregulated 10.5-fold in root and 5.3-fold in leaf of FL478 and 8.3- and 2.9-fold in Kamini (Figure 8A). No significant change in expression was observed in susceptible genotypes, except 2.5-fold increase in the roots of AC847. Similarly, the expression of *OsSOS2* and *OsSOS3* was also significantly induced in root and leaf of FL478 and Kamini, although the magnitude of induction differed (Figures 8B,C). But interestingly, the expression of *OsNHX1* showed significant upregulation only in Kamini, where it was 2.4 and 1.8 times upregulated in root and leaf, respectively (Figure 8D).

In this study, we examined the changes in expression of five other K^+^/Na^+^ transporters genes (*OsHAK5*, *OsAKT1*, *OsHKT1*, *OsHKT1;5*, and *OsHKT2;3*) belonging to three different classes. The expression of *OsAKT1* was downregulated in all the genotypes in response to salt stress, except FL478, where its expression did not alter as compared with control (Figure 8E). Another important potassium transporter belonging to the major K^+^ transporter family (KT) is *OsHAK5* expression, which was slightly increased in root of FL478 and in both root and leaf of Kamini. But it was downregulated in the IR29 and AC847 genotypes under saline condition (Figure 8F). The expression of *OsHKT1;5* (also known as *OsSKC1*/*OsHKT8*) was upregulated in leaves (7.1-fold) and in roots (3.6-fold) of FL478 (Figure 8G). Similarly, the induction was also significant in Kamini, where it was upregulated (2.2-fold) in the leaf under stress. Expression of another important Class I HKT family transporter *OsHKT1;1*, showed significant upregulation in the leaf and root of FL478 (∼1.8-fold) and in the leaf of Kamini (∼2-fold), whereas it was either unchanged or downregulated in other cases (Figure 8H). On the contrary, the expression of *OsHKT2;3*, a member of Class II HKT family transporters, was significantly high in root (2.8-fold) and leaf (∼2-fold) of FL478 (Figure 8I). We checked the expression of *OsROK*, an outward K^+^-rectifying channel. Interestingly, its expression was highly upregulated in leaf (3.5-fold) and root (3-fold) of IR29 and AC847 (2.8-fold in leaves and 4.4-fold in roots), whereas the induction was much less in both FL478 and Kamini (∼1.4–1.6 fold) (Figure 8J).

### Changes in the Transcript Abundance of Different Proton Pumps

Expression analysis of two plasma membrane (*OsAHA1* and *OsAHA7*) and two vacuolar H^+^ pumps (*OsV-ATPase* and *OsV-PPase*) revealed that expression of *OsAHA1* highly induced in FL478. It showed 5.8- and 2.9-fold upregulation in root and leaf, respectively (Figure 8K). Similarly, the expression of *OsAHA7* showed considerably higher upregulation in FL478, IR29, and Kamini (Figure 8L). On the contrary, both vacuolar H^+^-ATPase and pyrophosphatase (*OsV-ATPase* and *OsV-PPase*) showed significant upregulation in transcript abundance in leaf and root of Kamini (Figures 8M,N).

## Discussion

Often, soil salinity calls upon a plethora of impediments for plant growth and survival. Tolerance to salt stress is also governed by complex traits operating at physiological, biochemical, and molecular levels in plants. The excess Na^+^ present in the growing media causes several metabolic imbalances hindering normal growth. Among different complexities of stress tolerance mechanisms, maintaining an optimal cytosolic Na^+^/K^+^ ratio is still considered to be the most critical aspect of salt tolerance (Chakraborty et al., 2018). To maintain the optimal cytosolic Na^+^/K^+^ ratio in metabolically active tissues, plants need to perform highly energy-consuming processes of Na^+^ exclusion or sequestration coupled with cytosolic K^+^ retention or better uptake of K^+^ from an environment dominated by Na^+^. Again, all of these strategies are highly energy expensive, as they are mostly achieved by active transport of ions against the concentration gradient utilizing considerable amount of ATPs in the process (Rubio et al., 2019). Perhaps the energy cost of salt tolerance is so high that more often than not there is substantial yield penalty observed in salt-tolerant genotypes or in plants where different salt-tolerant traits were transferred (Cushman, 2001). In the present study, the genotype Kamini phenotypically manifested high salt tolerance, which was at par with FL478. But interestingly, the Na^+^-exclusion strategy did not solely condition such level of salt tolerance in Kamini. Rather, this genotype seemed to use some degree of Na^+^ as osmoticum to tolerate salt and osmotic stress owing to their superior tissue-tolerance ability. Coexistence of both ion-exclusion and tissue-tolerance strategies in same background and understanding their mutual contribution to salinity tolerance can be of immense importance for developing salt-tolerant rice cultivars with potentially lower energy cost for tolerance.

### Salt-Tolerance Rice Genotypes May Not Solely Depend on Na^+^-Exclusion and/or K^+^-Retention Strategies

Studies over the years revealed that several factors determine salt-tolerance ability in rice and other crops. Of them, tissue Na^+^/K^+^ ratio, biomass production, and chlorophyll pigment-retention ability under stress is still considered to be most critical for plant survival and stress tolerance (Munns, 2002; Moradi and Ismail, 2007; Kanawapee et al., 2012; Chakraborty et al., 2016a). In the present study, based on visual scoring and survival count, it was clear that both FL478 and Kamini were more salt tolerant than IR29 and AC847 at 12 dS m^–1^ of NaCl salt stress at early seedling stage (Figure 1). But salinity-induced reduction in root and shoot biomass and destruction of chlorophyll pigments were found to be the least in FL478, followed by Kamini, which suggests greater salt-tolerance potential of FL478 than Kamini. At the end of 7 days of stress, leaf chlorophyll content was almost double in these two genotypes as compared with that of IR29 and AC847. Interestingly, when we analyzed the tissue Na^+^ and K^+^ content in roots and shoots, it was observed that despite showing almost a similar phenotypic response under stress, FL478 and Kamini differed in uptake and accumulation of Na^+^ in roots and leaves. But there were not many differences in K^+^-retention ability either in roots or in leaves of these two genotypes. Both of them could manage to retain considerable tissue K^+^ content under stress as opposed to susceptible genotypes like IR29 and AC847 (Supplementary Figure S3). Previous studies also reported that tolerant genotypes could maintain less sodium and a low Na^+^/K^+^ ratio in upper plant parts and more importantly selectively transport potassium over sodium to maintain cellular ion homeostasis under salt stress (Moradi and Ismail, 2007; Guo et al., 2012; Chakraborty et al., 2019).

Our results clearly depicted that FL478 and Kamini employed different strategies to withstand the ill effects of salt stress yet achieved considerable salt tolerance. Further, when we checked the tissue Na^+^/K^+^ ratio and ability of ST of K^+^ over Na^+^ from root to upper parts, a marked difference in ST values between FL478, Kamini, and the other two genotypes was observed (Figure 2). It was quite evident that FL478 had much higher ST value right from the beginning of the stress period, which suggested maximum ionic-discrimination strategy operating in this genotype. After Na^+^ uptake, FL478 could able to restrict its upward movement from root to mesophyll tissues, perhaps owing to superior xylem unloading or vacuolar sequestration of Na^+^. On the contrary, Kamini, as compared with FL478, seemed to be much less selective in preferential upward ionic movement resulting in greater Na^+^ accumulation in leaves. It was observed that susceptible genotypes are unable to prevent accumulation of sodium in metabolically active mesophyll tissues, which often led to increased K^+^ depletion in upper plant parts (Reddy et al., 2014). Similarly, in the present study, both IR29 and AC847 showed higher Na^+^ accumulation and lesser K^+^ retention as reflected by much lower ST values and increased susceptibility to salt stress. Fluorescence tagging of Na^+^ by CoroNa green can reveal tissue-specific Na^+^ accumulation (Kutsuna and Hasezawa, 2005; He et al., 2015). In the present study, very high Na^+^ deposition around the vascular bundles of root in FL478 was noticed, which showed significantly higher green fluorescence intensity as compared with that of the other three genotypes, when stained with CoroNa green dye. The minimal changes in green fluorescence in the mesophyll tissues of FL478 under control and treated condition once again confirmed the ion discrimination and selective ion transport strategy operating in this genotype. But this classical ion-exclusion mechanism had significant deviation in another tolerant genotype, Kamini. This genotype had much less fluorescence intensity in roots but showed more fluorescence in the mesophyll tissue, which was comparable with that of IR29.

It is well-established that different Na^+^- and K^+^-specific transporters, *viz*., SOS, NHX, and HKT family transporters, play a crucial role in ion exclusion, Na^+^ sequestration, and *in planta* Na^+^ movement (Martinez-Atienza et al., 2007; Møller et al., 2009; Shabala and Munns, 2012). Similarly, transporters like HAK, AKT, KT, and KUP regulate K^+^ uptake and transport under saline condition (Fuchs et al., 2005; Nieves-Cordones et al., 2010). To have a clearer insight of differential Na^+^ accumulation and selective ion transport, we analyzed the gene expression profiles of some key Na^+^- and K^+^-specific transporters/ion channels in root and leaf tissues. Differences in the expression level of *OsSOS1*, *OsSOS2*, and *OsSOS3* between FL478 and Kamini in roots and leaves indicated the Na^+^-exclusion ability of these genotypes. Among the two, FL478 had higher expression, which supports its superior ion-exclusion capability over Kamini. This might be true as earlier works reported combined action of plasma membrane-bound SOS1, SOS2, and SOS3 transporters actively pump out Na*^+^* in rice under saline condition (Quintero et al., 2002; Guo et al., 2004). It would be clearer if we compare gene expression data with leaf Na^+^ content; however, Na^+^ accumulation pattern in root may not be explained by this. But if we consider the changes in the expression profiles of different Class I and Class II HKT transporters, then such response may be well-understood. The expression of *OsHKT1;5* (also known as *OsSKC1*) was very distinct in FL478 as compared with Kamini and other genotypes. The induction was almost four times higher than that of Kamini, which might be the reason for highly selective upward Na^+^ transport by FL478. Ren et al. (2005) reported *OsHKT1;5* as a strong candidate gene, predominantly works to unload Na^+^ from xylem stream and prevents upward transport of Na^+^. So the major mechanism of Na^+^ exclusion of FL478 might be xylem unloading of Na^+^ by hyperaction of HKT1;5 transporters present in the nearby xylem parenchyma cells. Perhaps owing to this, we could see high accumulation of Na^+^ near the vascular bundle of roots in FL478. As most of the absorbed Na^+^ is retained in the roots of FL478 due to high xylem unloading, hence, despite greater action of SOS1 transporter and considerable Na^+^ exclusion, still, this genotype accumulated almost a similar amount of Na^+^ as that of IR29 in the roots. But if we consider the net Na^+^ accumulation in both root and shoot tissue, then higher expression of *OsSOS1* transporter and overall Na^+^ exclusion mechanism can be justified. The *Saltol* quantitative trait locus (QTL) region was identified as one of the major QTLs governing early vegetative stage salt tolerance in rice, which acts to lower Na^+^/K^+^ ratio in shoots under stress (Bonilla et al., 2002). Later, *HKT1;5* or *SKC1* was identified as the major functional gene located inside this QTL region (Ren et al., 2005). Interestingly, Chattopadhyay et al. (2014) reported significant dissimilarity in the *Saltol* QTL region of Kamini from that of known salt-tolerant donor Pokkali and its derived line FL478. Some other salinity-tolerant landraces from the Sundarbans region such as Talmugur and Patnai also were found to be dissimilar from Pokkali in this QTL region. Thomson et al. (2010) also recorded a similar observation among salinity-tolerant lines in Saltol-QTL region. Variation in salt-tolerant alleles in *HKT1;5* gene could also explain the difference of two salinity-tolerant genotypes, FL478 and Kamini.

### Salt-Tolerant Genotype Kamini Balances Both Ion-Exclusion and Tissue-Tolerance Strategies and Might Spend Less Energy for Tolerance

Although both FL478 and Kamini were considerably tolerant to salt stress, a distinctive mechanistic difference in tolerance strategies in these two genotypes was observed. Kamini not only possessed less superior selective ion transport and Na^+^-exclusion ability than did FL478 but also accumulated higher Na^+^ in the mesophyll tissue. But in case of tissue K^+^ content, Kamini possessed at par K^+^-retention ability as FL478, which was superior than both IR29 and AC847. The expression data of *OsAKT1*, *OsHAK5*, and *OsROK* also supported this result. There was no or slight downregulation of *OsAKT1* in both FL478 and Kamini, whereas it showed higher downregulation in IR29. The activity of *OsAKT1* is highly sensitive to external Na^+^ condition, and it is reported to be downregulated under salt stress (Fuchs et al., 2005). Unlike *AKT1*, the Na^+^-insensitive K^+^ transporter *HAK5* plays a critical role as a high-affinity transporter responsible for K^+^ uptake in low K^+^ environment prevalent under saline condition (Nieves-Cordones et al., 2010; Yang et al., 2014). In the present study, upregulation in expression of *OsHAK5* was observed in tolerant genotypes, but not in IR29 under salt stress. The expression of *OsROK*, an outward K^+^ rectifying channel responsible for K^+^ leakage under salt stress, was highly upregulated in AC847 and IR29 whereas altered the least in FL478 and Kamini.

Tissue tolerance is a specialized trait, generally reported to be presents in salt-susceptible rice genotypes like IR29 and in some newly identified wild rice accessions (Prusty et al., 2018). The halophytic wild relative of rice, *Oryza coarctata*, was also identified to possess considerable tissue tolerance along with other important salt-tolerance traits (Mondal et al., 2018; Mangu et al., 2019). From our results, it is clear that besides K^+^ retention, Kamini must also possess considerable tissue-tolerance ability for maintaining integrity of chlorophyll pigment system under high leaf Na^+^ load. To test this, we performed leaf clip-based tissue-tolerance assay, where both Kamini and IR29 possessed much higher tissue-tolerance ability than known salt-tolerant genotype FL478. When we nullified ionic-discrimination ability by exposing the cut sections of the leaves directly to the NaCl solution, we found that FL478 required far less tissue Na^+^ to destroy half of the initial leaf chlorophyll content than did IR29 or Kamini (Figure 5). These data along with our results of chlorophyll fluorescence traits studied in the leaf clip assay suggested the existence of superior tissue-tolerance trait in Kamini.

Previous studies reported that during salinity stress, synthesis of osmo-regulators was the highest in tolerant genotypes to maintain favorable plant water status inside cells (Karthikeyan et al., 2011; Li et al., 2011; Zang et al., 2011). Interestingly, when we checked the contents of most common organic osmolytes, significantly higher accumulation of trehalose, proline, and GB was found in FL478, but in Kamini, it was at par with susceptible genotypes. At the same time, the drop in RWC and LWP was higher in IR29 and AC847, whereas it did not alter much in FL478 and Kamini. This is suggestive of potential ability of Kamini to use Na^+^ as osmoticum and avoid salinity-induced drop in plant water potential to maintain suitable plant water status. It could possibly reduce the burden of higher organic osmolyte biosynthesis in Kamini, whereas FL478 needed to synthesize greater amount of organic osmolytes at the expense of huge energy and C-skeleton.

### Differential Induction of H^+^ Pumps Suggests Relative Importance of Two Salt-Tolerance Strategies in FL478 and Kamini

Reduction in membrane potential of plasma membrane is most common in plants in response to salt stress (Bose et al., 2015; Chakraborty et al., 2016c). Rapid induction of plasma membrane and vacuolar proton pumps to maintain negative membrane potential and to facilitate Na^+^/H^+^ pumps was reported in salt-tolerant genotypes under stress (Vera-Estrella et al., 2005; Shabala and Mackay, 2011). Both Na^+^ exclusion and K^+^ uptake from rhizospheric region and selective upward transport mediated by xylem unloading is energy dependent and supported by increased action of ATPases and pyrophosphatases (Chakraborty et al., 2018; Niu et al., 2018a; Huang et al., 2019). To date, there are 11 variants of *AHA* (a plasma membrane ATPase) reported in rice (Ueno et al., 2005). Among them, a few are reported to express in both root and shoot tissue to generate electrochemical gradient for nutrient uptake and its further transport (Haruta and Sussman, 2012). Both AHA1 and AHA7 were reported to be responsible for chemiosmotic movements of ions, whereas AHA7 is primarily associated with root hair development and nutrient uptake (Kumar et al., 2015; Yuan et al., 2017). In the present study, upregulation of different plasma membrane H^+^ pumps (*OsAHA1* and *OsAHA7*) was observed in roots and leaves of studied genotypes in response to salt stress (Figure 8). Interestingly, there was significant upregulation of vacuolar H^+^ pumps (both *V-ATPase* and *V-PPase*) under salt stress. But distinctively, plasma membrane H^+^ pumps were more upregulated in FL478, whereas vacuolar H^+^ pumps showed greater induction in Kamini. Highly energy demanding Na^+^ exclusion by the action of either SOS1 or HKT group of transporters requires active pumping of H^+^ against the concentration gradient to maintain favorable plasma membrane potential under stress (Sze et al., 1999; Maeshima, 2000; Morsomme and Boutry, 2000; Ratajczak, 2000). Hence, it is quite natural that a genotype like FL478 showed higher induction of plasma membrane H^+^ pumps to support their predominant Na^+^-exclusion strategy as evident from changes in transcript abundance of different Na^+^- and K^+^-specific transporters. On the contrary, a genotype like Kamini having lesser ionic-discrimination and Na^+^-exclusion capacity depends more on tissue-tolerance ability. Perhaps, this reaffirms our hypothesis that a genotype like Kamini was more keen to accumulate greater Na^+^ under salt stress and managing the Na^+^ load by effective vacuolar sequestration.

## Conclusion

Taken together, the present study pointed out interesting mechanistic differences in two rice genotypes, both of which were tolerant to salt stress yet employed different strategies to withstand stress. The physiological (biomass production, chlorophyll retention, tissue Na^+^ and K^+^ content, and selective ion transport ability) and molecular (expression analysis of different Na^+^ and K^+^ transporter/ion channels) evidences suggested that salt-tolerant genotype FL478 might be a good Na^+^ excluder and had high ionic-discrimination ability, which resulted in reduced transport of Na^+^ to upper plant parts and helped in maintaining a very low Na^+^/K^+^ ratio in the leaves. On the other hand, another salt-tolerant genotype Kamini did not possess as good ionic selectivity, which resulted in a higher Na^+^ content and an increased Na^+^/K^+^ ratio in the leaves. But besides having moderate ion-exclusion capacity, Kamini, as compared with FL478, possessed very good tissue-tolerance ability (as evidenced from our tissue-tolerance assay). The results of our study indicated that the two components of salt tolerance mechanism, that is, ionic selectivity and tissue tolerance, are very distinct in nature, and their coexistence is probably independent of each other. A genotype like Kamini could achieve considerable salt tolerance by effectively balancing both ion-exclusion and tissue-tolerance abilities, which might give a new insight toward minimizing the energy cost of salt tolerance. The mechanism employed by Kamini could probably save energy for salt tolerance in two ways: (i) reducing the energy cost of ionic selectivity and ion exclusion by active pumping out Na^+^ either during uptake process or during upward transport (xylem loading) and (ii) owing to its superior tissue-tolerance ability, it could use some amount accumulated Na^+^ as osmoticum, thereby reducing the need of highly energy-utilizing organic osmolyte biosynthesis process. Hence, the present study gives us an insight on ideal salt-tolerance strategies needed for rice to survive under high saline environment to minimize the energy cost of tolerance. This would be particularly useful for future rice crop improvement program for salinity tolerance, where there is a need to decide whether we should focus on imparting highly energy-requiring ion-exclusion strategy or on genotype like Kamini where both ion-exclusion and tissue-tolerance strategies are effectively balanced, which could potentially minimize the energy cost of salt tolerance.

## Data Availability Statement

All datasets generated for this study are included in the article/Supplementary Material.

## Author Contributions

KoC, RS, and SR conceived the study and designed the experiment. SM and BP conducted hydroponic and tissue-tolerance assays, and physiological studies. KoC, SR, and PSa did all the molecular studies. KoC and SM analyzed the data and did statistical analysis. KoC, SM, SR, and RS wrote the manuscript. KrC, MK, and PSw gave valuable suggestions and did necessary revision in the manuscript. All the authors read and approved the manuscript.

## Conflict of Interest

The authors declare that the research was conducted in the absence of any commercial or financial relationships that could be construed as a potential conflict of interest.

## Footnotes

1 www.quantprime.de

## Abbreviations

ChlF: chlorophyll *a* fluorescence
DW: dry weight
EC: electrical conductivity
F_m_: maximum fluorescence
FW: fresh weight
GB: glycine betaine
LC_50_: a sodium concentration where half of the chlorophyll pigments were degraded
LWP: leaf water potential
RWC: relative water content
SES: standard evaluation score
SOS: salt overly sensitive
ST: selective transport of potassium over sodium
TW: turgid weight
VSI: visual salt injury
Y(II): efficiency of photosystem II
Y(NO): quantum yield of non-regulated energy dissipation.
