# Supplementary material for: Tissue Tolerance Coupled With Ionic Discrimination Can Potentially Minimize the Energy Cost of Salinity Tolerance in Rice
Source: Front Plant Sci. 2020 Mar 25;11:265. doi: 10.3389/fpls.2020.00265 (PMC7109317; doi:10.3389/fpls.2020.00265)

Supplementary Table S1. Details of the primers used for real time q-PCR study for expression analysis of studied genes with their MSU ID/Gene Bank Accession Number

| **Gene Name** | **MSU ID/ Gene Bank Acc. No.** | **Primer Name** | **Sequences** |
| --- | --- | --- | --- |
| *OsSOS1* | LOC_Os12g44360.1 | *OsSOS1_*F | AGATCGCGCTTACTCTTGCTGTC |
|  |  | *OsSOS1_*R | AGACCTCCAGTGCATCTTGTGC |
| *OsSOS2* | LOC_Os06g40370.1 | *OsSOS2_* F | ACTTAGCACTTTGGCCCAGAAAG |
|  |  | *OsSOS2_* R | ACCACATGACCAAACATCTGCTG |
| *OsSOS3* | LOC_Os05g45810.1 | *OsSOS3_* F | GAACATGTCACTTCCCTATTTGC |
|  |  | *OsSOS3_* R | GTCATGGGCTTCTGAATGCATT |
| *OsNHX1* | LOC_Os07g47100.1 | *OsNHX1_* F | TGACCGTGAGGTTGCCCTTATG |
|  |  | *OsNHX1_* R | GAGAATGCCGCTCAAATCTAGCAA |
| *OsAKT1* | LOC_Os01g45990.1 | *OsAKT1_* F | GGAGCTGATCCAAATGCCAGAG |
|  |  | *OsAKT1_* R | TGCAAGCGTATAAGCCCGTGTC |
| *OsHAK5* | LOC_Os01g70490.1 | *OsHAK5_* F | TCGCATCTATCCAGAACACGTTGC |
|  |  | *OsHAK5_* R | TATGCATTGCCGATCTTGTCTGTAG |
| *OsHKT1;1* | LOC_Os06g48810.1 | *OsHKT1;1_* F | GGCGTTTCTGGCATCAACTGTC |
|  |  | *OsHKT1;1_* R | ATTCCAGTCGACAGCACCGAAC |
| *OsHKT1;5* | LOC_Os01g20160.1 | *OsHKT1;5_* F | GTCGTGCTCTACGTGGTGATG |
|  |  | *OsHKT1;5_* R | CTCCCGTTTGCTGGTGTTTGTC |
| *OsHKT2;3* | LOC_Os01g34850.1 | *OsHKT2;3_* F | AACATCATCTTTGAGGTGATAAG |
|  |  | *OsHKT2;3_* R | AAGTTGTACGCCTTCTCATGGC |
| *OsROK* | AB248822.1 | *OsROK_* F | CACCCAAGTACAAATAGCAAAGG |
|  |  | *OsROK_* R | CTGCACAGGGAGTTTCTTT |
| *OsAHA1* | LOC_Os03g48310.1 | *OsAHA1_* F | ACAGAACCTGGCTTGAGTGTG |
|  |  | *OsAHA1_* R | GGGCAAGCAGCATAAACCCAAA |
| *OsAHA7* | LOC_Os04g56160.1 | *OsAHA7_* F | GGAGATCAAGAATGAGGCCG |
|  |  | *OsAHA7_* R | CTCCTCGATCGGTATGTTCTC |
| *OsV-PPase* | LOC_Os06g08080.1 | *OsV-PPase_* F | ATGGCTCTCTTCGGAAGGGTTG |
|  |  | *OsV-PPase_* R | GTCACCGACATTGTCAGCAATCAC |
| *OsV-ATPase* | LOC_Os11g06890.1 | *OsV-ATPase_* F | ACTACCTCTTTGACGGCTACGC |
|  |  | *OsV-ATPase_* R | GCTTTGGTTGCTGTGCATTTGCC |
| *Os18SrRNA* | AK059783 | *Os18SrRNA*_F | ACATAGAAGGAGAAGAATGCACCCGC |
|  |  | *Os18SrRNA*_R | ACACTTCACCGGACCATTCAA |

Supplementary figure S1. Showing the detailed images of visual salt injury (VSI) to identify tolerance/susceptible level of different rice genotype under salt stress.


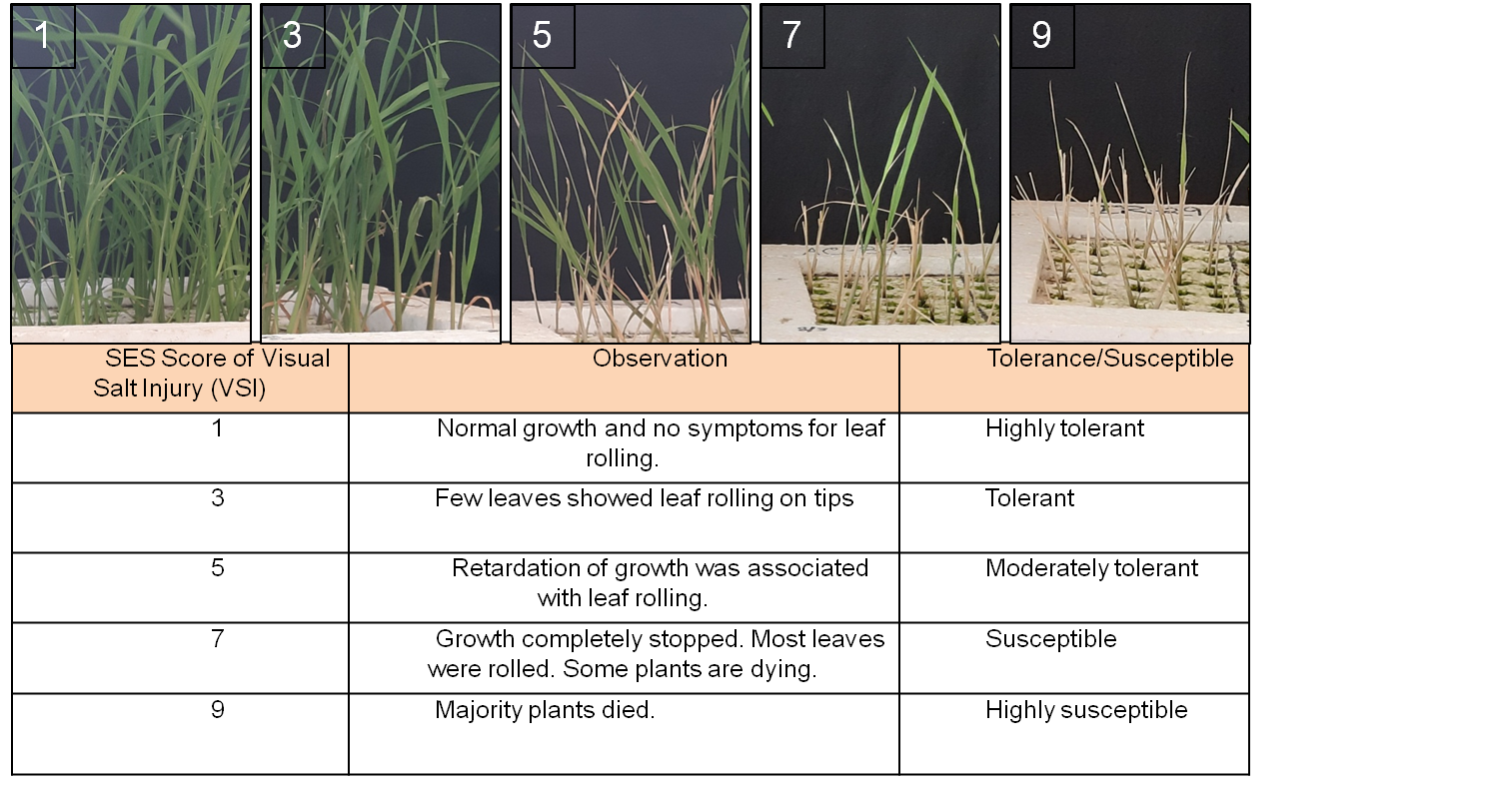


Supplementary figure S2. Effect of salt (12 dS m^-1^) on the fresh weight of root (A) and shoot (B); length of root (C) and shoot (D); and changes in chlorophyll content (E-H) in four rice genotypes subjected to seven days of stress in hydroponic assay. The values presented are the mean ± SE of five independent biological replications.


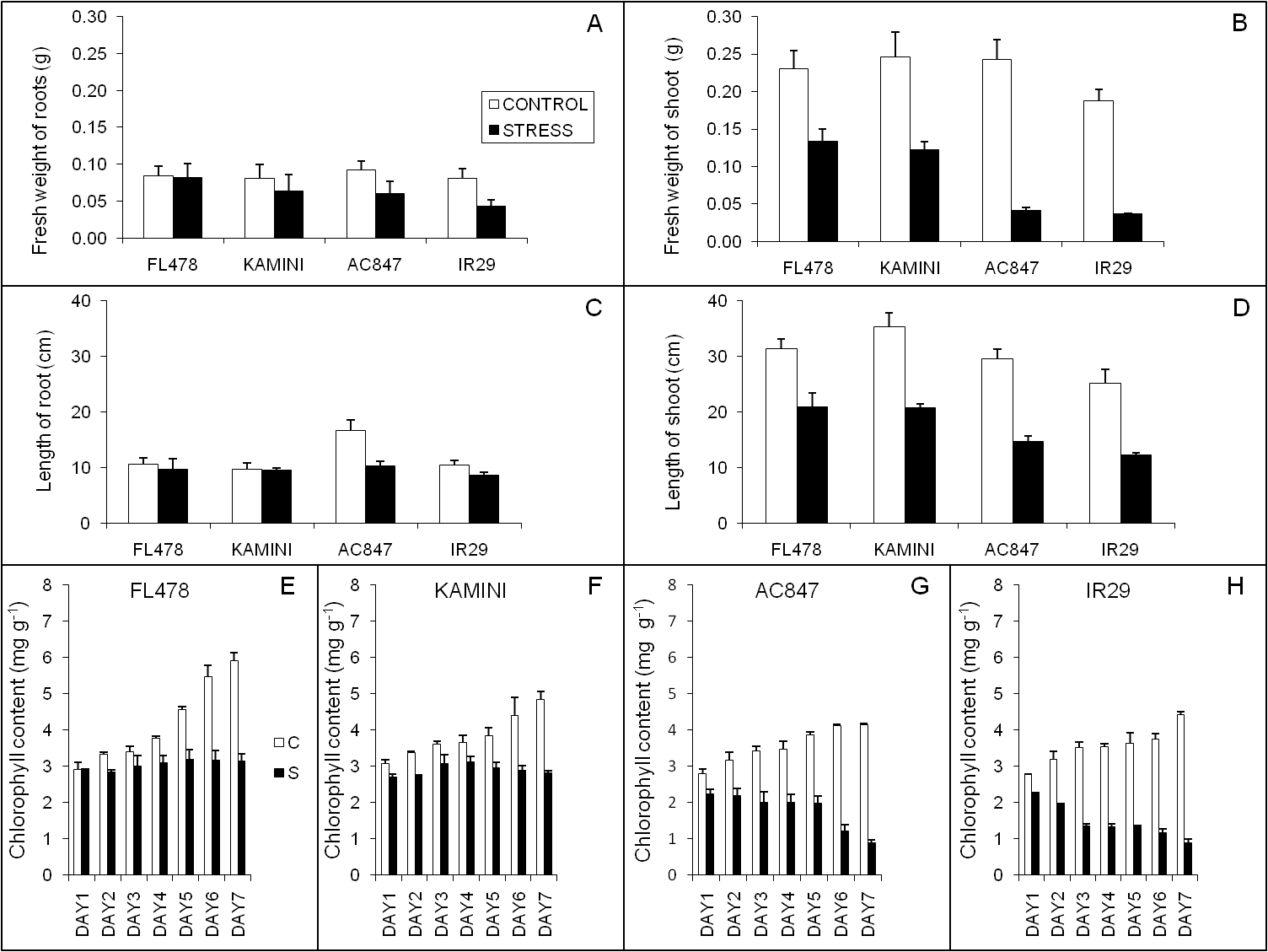


Supplementary figure S3. Effect of salt (12 dS m^-1^) on day-wise root and shoot Na^+^ (A) and K^+^ (B) accumulation in four rice genotypes subjected to seven days of stress in hydroponic assay. The values presented are the mean ± SE of five independent biological replications.


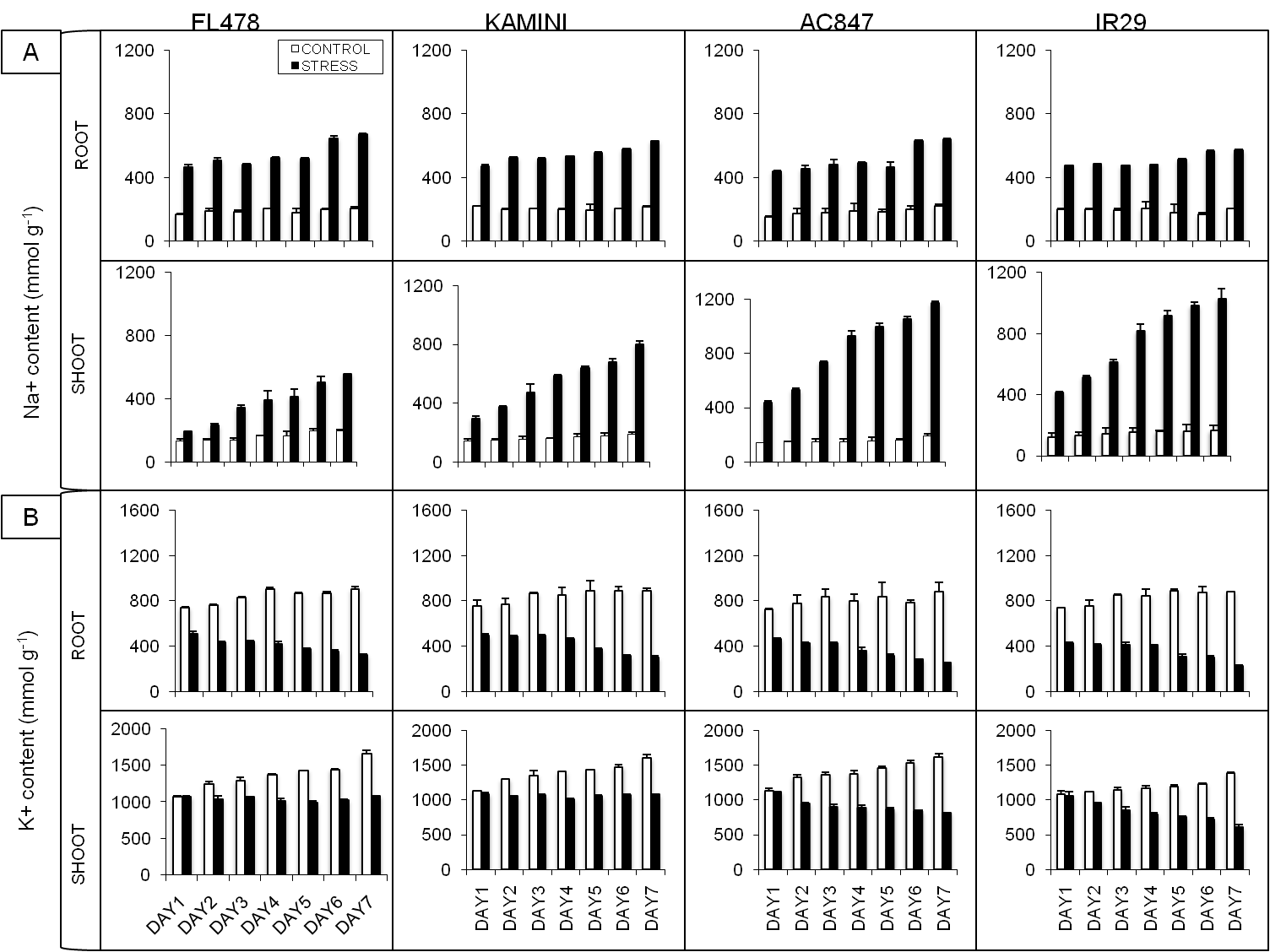


Supplementary figure S4. Showing the detailed images of root sections stained with CoroNa Green and Propidium iodide (PI) both control and stressed condition of four rice genotypes, where A represents FL478, B represents Kamini, C represents AC847 and D represents IR29 genotype.

Supplementary figure S5. Showing the detailed images of leaf sections stained with CoroNa Green and Propidium iodide (PI) both control and stressed condition of four rice genotypes, where A represents FL478, B represents Kamini, C represents AC847 and D represents IR29 genotype.


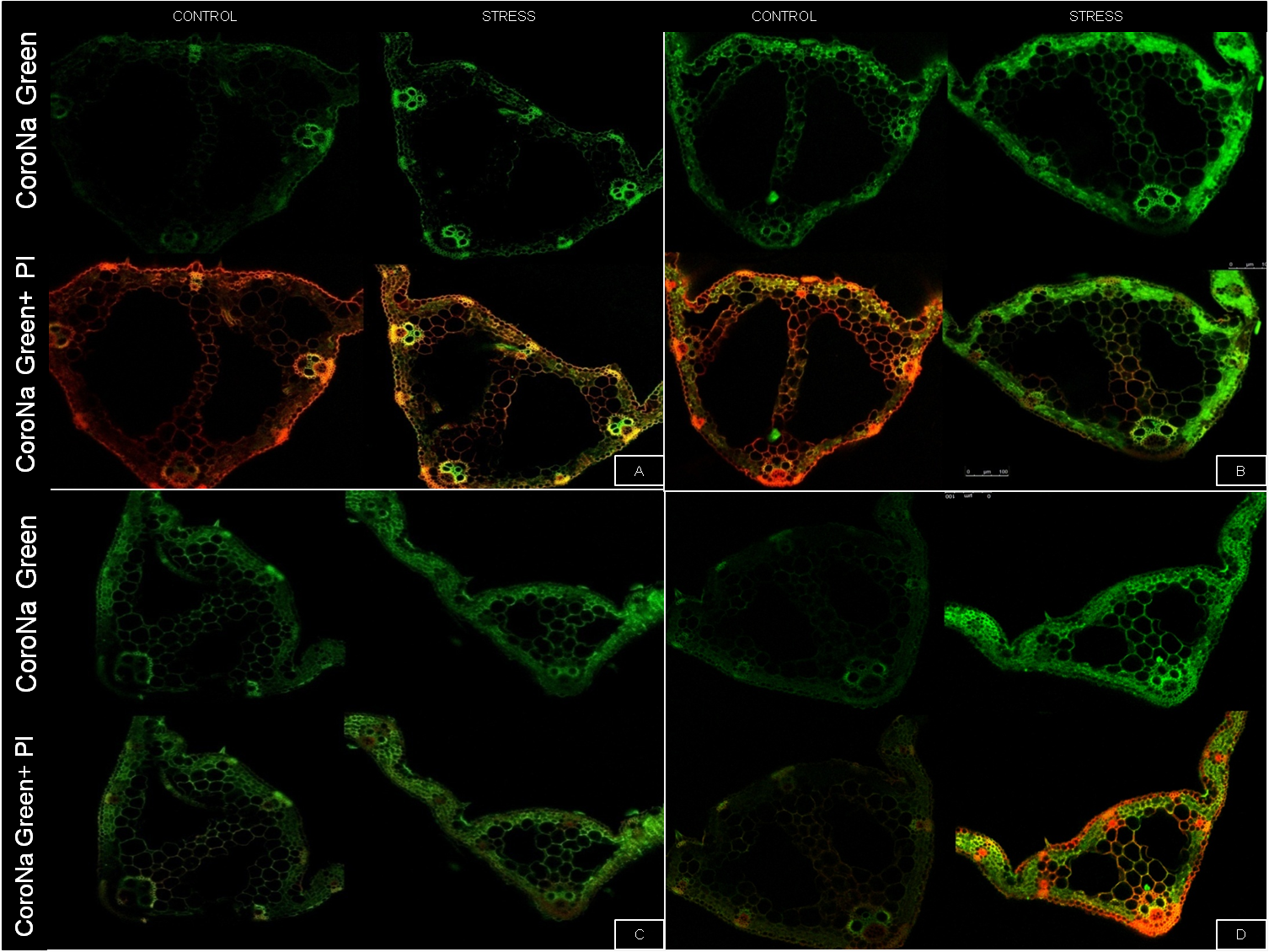

Supplement: Supplementary file 1 [file Data_Sheet_1.docx]
